# Partition and Poliomyelitis: An Investigation of the Polio Disparity Affecting Muslims during India's Eradication Program

**DOI:** 10.1371/journal.pone.0115628

**Published:** 2015-03-05

**Authors:** Rashid S. Hussain, Stephen T. McGarvey, Lina M. Fruzzetti

**Affiliations:** 1 Warren Alpert Medical School of Brown University, Providence, RI, United States of America; 2 Brown University Department of Epidemiology, International Health Institute, Providence, RI, United States of America; 3 Brown University Department of Anthropology, Brown University, Providence, RI, United States of America; Columbia University, UNITED STATES

## Abstract

**Background:**

Significant disparities in the incidence of polio existed during its eradication campaign in India. In 2006, Muslims, who comprise 16% of the population in affected states, comprised 70% of paralytic polio cases. This disparity was initially blamed on the Muslims and a rumor that the vaccination program was a plot to sterilize their children. Using the framework of structural violence, this paper describes how the socio-political and historical context of Muslim populations in India shaped the polio disparity.

**Methods and Findings:**

A qualitative study utilizing methods of rapid ethnography was conducted from May-August 2009 in Aligarh, Uttar Pradesh, India. Field methods included participant observation of vaccination teams, historical document research, and 107 interviews with both Global Polio Eradication Initiative (GPEI) stakeholders and families with vaccine-eligible children. Almost all respondents agreed that Aligarh was a highly segregated city, mostly due to riots after Partition and during the 1990s. Since the formation of segregated neighborhoods, most respondents described that "Muslim areas" had been underdeveloped compared to "Hindu areas," facilitating the physical transmission of poliovirus. Distrust of the government and resistance to vaccination were linked to this disparate development and fears of sterilization influenced by the "Family Planning Program" from 1976-1977.

**Conclusions:**

Ethnic violence and social marginalization since the Partition and during the rise of Hindu nationalism led to distrust of the government, the formation of segregated slums, and has made Muslims victims of structural violence. This led to the creation of disease-spreading physical environments, lowered vaccine efficacy, and disproportionately higher levels of resistance to vaccination. The causes of the polio disparity found in this study elucidate the nature of possible other health disparities affecting minorities in India.

**Limitations:**

This study is limited by the manual coding of the transcribed data, size, and some dialectal difficulties in translation.

## Historical Background and Theoretical Basis of Study

### General

In 1988, the World Health Assembly declared that polio would be eradicated by the year 2000. With the use of the Oral Polio Vaccine (OPV), the Global Polio Eradication Initiative (GPEI) has decreased the incidence of paralytic polio by more than 99%, and eradicated the P2 strain of the virus [1]. Though the remaining strains of the poliovirus (P1 and P3) are now also eradicated in India, they disproportionately affected the Muslim community until recently [2]. In 2006, 70% of polio cases in Uttar Pradesh (UP) came from the Muslim community, which comprised only 16% of the state’s population [3, 4]. In the popular press, this disparity was blamed on ‘stubbornness’ and ‘ignorance’ on the part of the Muslims who believed the vaccination program was a plot to sterilize their children [5]. Generalizations such as these ignore local realities including the history of India’s Muslims as a “minority” community and the structural and environmental factors that rendered Muslims more susceptible to the spread of polio.

### Historical Background

With the 1947 Partition of British India into Pakistan as a “Muslim homeland” and India as a secular, but, “Hindu-majority” state, the Muslims of India found themselves the legacy of a divided nation. Seven million Muslims left for Pakistan and over a million people died in the violence that ensued [6]. Muslims were historically blamed for the violence because many perceived them to have caused the Partition of India. Additionally, their patriotism often became suspect when relations between Pakistan and India soured, as many saw them as inherently more loyal to their co-religionists in Pakistan [7].

These antagonistic feelings—a legacy of colonialism—fueled the continued rise of Hindu nationalism in the post-Partition era. The Hindu nationalist movement was one which defined a true Indian as a Hindu who regarded India as his or her fatherland, motherland, and holy land [7]. With this ideology, Muslims, and to a certain extent Christians, were excluded from the national fold. These ideas often became embedded in the psyche of individuals in areas which saw religious conflict most, leading to distrust and discrimination between communities. For example, when Sanjay Ghandi of India’s secular Congress party launched the “Family Planning” program which promoted sterilization as a means of population control, Muslim groups felt particularly targeted [8]. Though the policy was not supposed to be discriminatory based on religion, certain incidents—like one in Uttawar, Haryana, where young Muslim men were dragged from their homes in the middle of the night and given the choice to either get sterilized or have illegal weapons possession charges levied against them—indicate that the perceived discrimination may not have been completely unfounded [9]. Feelings of both physical and social exclusion through increased ghettoization ensued in the following decades. This was especially true in the North Indian States like Uttar Pradesh and Bihar where the legacy of Partition with split families and a disaffected Muslim population were strongest [10].

Indian Muslim sentiments regarding feeling “targeted” by their government were exacerbated when fertility politics were employed during the rise of Hindu nationalist political parties in the 1990s. During this time, politicians attempted to rally a Hindu voting base by exploiting fears that Muslims, who already divided the country and “had a country of their own” would out-populate Hindus in “their own country.” For example, Hindutva politicians in the city of Aligarh claimed that Muslims in peripheral slums were proliferating more rapidly than the Hindus, and that Hindus would soon be outnumbered [11]. During the 1990–1991 riots, communal violence was concentrated in these stigmatized slums, speculated to have been targeted due to their supposedly high birthrate [11]. Communal tension peaked during this era with the destruction of the Babri Mosque in 1992, after which the country experienced the worst riots since the Partition [7, 10, 11]. It was during this tumultuous era of distrust and disharmony that the polio eradication program was started by the Government of India as part of the GPEI [12], shortly before a Hindu nationalist coalition government formed in May of 1995.

Since that time, work has been done documenting higher rates of relative deprivation among Muslims in India. In 2006, the Government of India published the Sachar Committee Report which explained how Indian Muslims had fallen behind other minorities with regard to both education and economic status [13]. Additional studies since the Sachar report have corroborated its findings and called for a more comprehensive understanding of the realities facing the Muslim community [10, 14].

Neither Muslims nor Hindus in India are homogenous groups: they encompass a diversity of customs, socioeconomic status and cultural beliefs. The groups often have more diversity within their community than between them, with some syncretic forms of Islam resembling the local Hindu tradition more than the orthodoxy, and some self-identified Hindus sometimes holding traditionally Muslim names and beliefs [6, 7, 10, 11, 15]. Class and caste continue to be major divisors amongst both Hindus and Muslims, often being a more important differentiator than religious community.

The history of the region suggests that Muslim disenfranchisement is inextricable from general ideas of social class. Muslims from wealthier classes and families interacted very little with their co-religionists both historically and contemporarily. Wealthier residents in Aligarh’s Civil Lines rarely, if ever, cross over the railway tracks to the old city where the city’s impoverished urban Muslim population predominantly lives [16]. The Muslim League, the political party that led the movement for the formation of the Pakistani state, was considered a distanced and exclusionary group of Northern landed elite Muslims for much of its history, and even later got most of its support in particular North Indian regions [17]. This group, often known as the “sharif” or “ashraf” elite of the North Indian Muslims had in effect started speaking on behalf of all of the Muslims, including the lower class “ajlaf” Muslims. With Partition, mostly the upper and middle classes among the Muslims fled to Pakistan, leaving a more vulnerable population behind [18]. A similar parallel continued to play out in Aligarh during the 1990s when the city had become one of the most riot-prone in the country, with Hindu nationalists using the fact that the Aligarh Muslim University was a center of the Pakistan movement as a rallying cry. Whenever the riots occurred, they usually spared the Civil Lines where the university was and affected the poor urban Muslims who had little to no connection with either the university or its history [11, 17]. This violence parallels how the masses of poor Indian Muslims are often targeted whenever there is anti-Pakistan sentiment despite the poorer Muslims’ generally weak connections to both the historic Pakistan movement and the modern Pakistani state.

Notably, these legacies are perhaps weakest in South India where Muslims were least impacted by either Partition or the Hindu Nationalist movement, and are notably less socially marginalized. Additionally polio was not considered endemic there at the time of the study. In some ways, the broad marginalization of Indian Muslims can be seen as a marker for both the larger size of the Muslim population in the Northern states which are poorer and less developed than the rest of the country [10]. It is important to note that apart from the Urdu-speaking North Indian Muslims, there are a wide variety of Indian Muslims: from the Mappilas of Kerala, to the trading clans of Gujarat like the Bohras and Khojas, to the Konkans along the western coast, and the Bengali Muslims as well. With varied languages, customs, and modes of practice, Indian Muslims, let alone Hindus, can hardly be considered a monolith [19], though their religious and national identity binds them in a common narrative. That narrative continues to be predominantly that of the North Indian Urdu-speaking Muslims, who are the focus of this study, as they epitomize the legacy of Partition. The intricacy of the formation of Muslim and Hindu identities even in the North along with their modern manifestations is a highly elaborate topic which cannot be explained justly within the limitations of this paper. Nonetheless, despite the plural nature of Indian society, the legacy of colonialism, Partition, and rise of right-wing Hindu ideologies has rendered Muslims a sociological minority overall, especially in Northern Indian states like Uttar Pradesh where these legacies were strongest [7, 10, 11, 13, 17].

### Theoretical Basis

In the United States, historically marginalized populations such as African-Americans and Native-Americans suffer disproportionately from chronic and infectious disease [20]. These disparities occur because of “arrangements embedded in the political and economic organization of the social world,” which have been popularized in the theory of structural violence [21]. In contrast to the highly visible nature of physical violence, structural violence involves structures, institutions, and ideologies that are often hidden in plain sight or taken for granted. According to the theory, these forces enact violence on populations by disenfranchising them from education, employment, and healthy living, reducing upward mobility and quality of life indicators. The effect of these intangible forces can also be understood through the concept of embodiment, as described by Krieger (2005), where physical manifestations, such as disease, show the story of the affected individuals [22]. In such circumstances, we would thus witness the embodiment of structural violence as trajectories of marginalization lead to disproportionate disease burdens. These complementary theories provide a framework for identifying the specific pathways through which historic and social factors of marginalization become manifest in the bodies of marginalized populations. Notably, structural violence has been used to describe how legacies unique to the Indian Muslim contributed to a new collective Muslim identity as an “other” within Indian society, though the health consequences of this were not considered [23].

The association between structural violence and adverse health outcomes among minority populations is well-documented. This includes both objective measures of poverty and residential segregation as well as measures of perceived discrimination and relative deprivation [24, 25, 26, 27]. In particular, self-reported discrimination among minority populations has been associated with decreased participation in healthy behaviors and utilization of health services [26, 27]. It should be noted that these measures are multidimensional concepts that lead to increased vulnerability through multiple biological and social pathways. These can be manifest as compromised stress responses, mistrust in health systems, or in other ways depending on the local context.

The contributors of social marginalization additionally align with concepts used to understand patterns of vaccination behavior. Streefland et al (1999) explain that understanding vaccination acceptance and non-acceptance relates to population trust in systems, providers, and perceptions of risk and quality, especially if vaccination is seen as a form of state power and control [28]. While no one pathway can explain vaccine behavior, applying the factors of structural violence to current vaccine behavior explanatory frameworks is useful in understanding the causal pathways for vaccine behavior as it relates to disparities affecting Indian Muslims.

### Aims of Paper

This project was conducted to understand the sociopolitical and historical dimensions of the polio disparity between Muslims and Hindus in India. It builds on previous work highlighting deprivation among Indian Muslims, using the theory of structural violence to systematically analyze the pathways through which Muslims’ marginalization increased their susceptibility to polio in addition to factors of vaccine non-acceptance.

## Methodological Approaches to the Research

### Ethics Statement

Proper informed consent was taken for all interviews and financial compensation provided, in accordance with a protocol approved by the Brown University IRB, and the medical administration at Jawaharlal Nehru Medical College. Both verbal and written consent was obtained from literate respondents who were provided a copy of the consent documents for their records. Only verbal consent was obtained if subjects were not literate, though they were provided a copy of a separate consent document for their records. All consent forms and procedures were approved by the IRB and Jawaharlal Nehru Medical College.

### General Protocol

This research took place in the city of Aligarh in Uttar Pradesh, India. Aligarh District was classified as a “high-risk” district for the spread of polio by the GPEI [29] and was the source of the majority of India’s polio cases in 2003 [30]. Home to the Aligarh Muslim University (AMU) that played a key role in the Pakistan movement, the city has seen frequent riots over the years [11, 16]. The situation in Aligarh and the stigma faced by its Muslim population set the social and historical context for the conditions that, this paper argues, caused the polio disparity.

The methods were conducted in accordance with the principles of rapid ethnography/rapid assessment procedures (RAP) and included in-depth key informant interviews, behavioral observation, and semi-focus groups [31]. The qualitative data was collected over four months, May—August 2009. Methods included participant observation of two clinics operated by AMU as part of the Underserved Strategy with support from Unicef and Rotary International, in addition to three week-long door-to-door polio rounds, interviews with 27 stakeholders in the polio program, and 80 semi-structured interviews with families who interacted with the polio program. Informal interviews were embedded within the participant observation while the structured interviews were conducted afterward.

### Theoretical Framework

We used rapid ethnography to understand local perceptions, attitudes, values, and experiences with illness and violence that might not be captured by more quantitative approaches [31–32]. Rapid ethnography also offered advantages over traditional ethnography which would have taken longer for a more in-depth analysis of the social realities at the cost of timely advice, information [31]. An ethnographic approach allowed explanations which would otherwise have been obscured by cultural reasons or blaming, facilitating a nuanced exploration of factors influencing differential participation in treatment programs [33].

### Participant Observation and Unstructured Interviews

The researcher used the method of participant observation to collect data both at clinics run by the GPEI and the door-to-door vaccination program. During the door-to-door program, the researcher was embedded with polio vaccination teams as they attempted vaccinating families who had actively refused in the past to accept vaccination, described as “resistant” families. Three polio rounds were conducted during the course of the study, preceded by booth days which started on May 26, July 5, and August 9 of 2009. During the rounds, the researcher was perceived to be a part of the vaccination team, and held onto charts and paperwork while observing interactions between the vaccinators and the families. Field notes were taken of families’ reaction to the polio program, health conditions in the neighborhoods, and the behavior of the information, education and communication (IEC) teams of the Social Mobilization Network (SMNet) which consisted of UNICEF Community and Block Mobilizing Coordinators (CMCs and BMCs)—who oversaw the operation and served as gatekeepers between the medical system and the community by engaging with community leaders and families about the program—[34] as well as medical interns from the Jawaharlal Nehru Medical College and Ajmal Khan Tibbiya College. Participant observation of the GPEI-run clinics included noting interactions between the patients and staff, and conducting 15 informal interviews with clinicians. The participant observation of the polio rounds was used to gain a stronger understanding of the realities of vaccination on the ground, brainstorm challenges to the program, and compare the local situation to that expected from the initial literature review to fit within the theoretical framework of ‘structural violence,’ ‘embodiment,’ and health justice.

Participant observation at the GPEI pediatric clinic was conducted to provide insight into local health care and needs, while observation of the door to door Pulse Polio rounds provided insight into both workings of the program and families’ perception of it. A total of 22 informal interviews were also conducted with the vaccination teams during the course of data collection, and written alongside the field notes. The participant observation was also used to generate further research questions, and determine proper sites for the bulk of interview recruitment that occurred afterward. The participant observation stage of the study was not used for active participant recruitment itself.

### Active Recruitment and Structured Interviews

Twenty-seven formal interviews were conducted using in-depth, semi-structured interview guides after active participant recruitment with both grassroots and administrative stakeholders in the polio eradication program. The in-depth structured interviews were conducted with health promoters called Community Mobilizing Coordinators (CMCs) (n = 5), polio booth-workers (n = 5), clinicians who worked in underserved areas (n = 4), medical interns (n = 5), community physicians (n = 5), and administrators with the GPEI (n = 3). These diverse ‘stakeholder’ views were conducted to get an insight into how resistance was viewed by those involved with the eradication effort at various levels, and how they saw or sought to address the shifting nature of resistance.

Interview questions were based on data collected from the initial participant observation, and included questions about individuals’ knowledge and opinions about the polio eradication program, the oral polio vaccine, causes of “resistance” to vaccination, confidence in the program’s ability to succeed, and opinions about current or alternative eradication strategies. Interviews also included a survey of the city which encompassed demographics, and general perceptions of the populations regarding how it was organized socioeconomically, and why.

Eighty formal, semi-structured interviews were also conducted with families with children who interacted with the polio eradication program in major parts of Aligarh after active recruitment. Individuals from each family were interviewed based on their willingness to participate. All respondents were either the head or co-head of the family as mothers (n = 37) and fathers (n = 43) participated about equally. Though interview questions were geared towards one interviewee, if other family members contributed to the discussion, the interview was allowed to take its course as a semi-focus group. Other than one day of interviews where five “resistant” families were specifically sought out for interview in Jeevangarh, families with children were selected randomly. Participants were recruited by knocking on doors in the major streets/alleys of each ward as determined by neighborhood informants, asking for families with children who would be willing to interview until a total of around five families were interviewed in each ward as demarcated by local GPEI partner organizations. This occurred in all wards except Jeevangarh that had a total of nine interviewees, four random, and five exclusively “resistant” as described. This partially random selection yielded a diverse number of participants, including several who were “resistant” to vaccination. The number of interviews in each of the wards was Maulana Azad Nagar (n = 5), Jamalpur (n = 5), Civil Lines (n = 3), Jeevangarh (n = 9), Begambagh (n = 5), Devatray (n = 5), K.R. Jain (n = 5), Gandhi Nagar (n = 5), Upper Kot/Upper Fort (n = 5), Bhojpura (b = 6), Shahjamal (n = 6), Indira Nagar (n = 5), Bannadevi (n = 5), PPC (n = 5), and Mehfooz Nagar (n = 6).

These wards represented major blocks of population divided along socioeconomic and religious lines. This sampling was thus sought to get a broad overview of opinions in the community about the polio program, with “resistant” views being well represented. This was why “resistant” individuals were initially sought, though “resistance” was found to be common enough that neighborhood opportunity sampling yielded resistant families who had interacted with the program. Of all who gave their informed consent to participate in the study, 77 families continued the semi-structured interviews to completion. A total of 3 families from Bhojpura, Jeevangarh, and Mehfooz Nagar decided to stop the interview midway for an unspecified reason. Participants were asked questions about their knowledge and opinions about the polio eradication program, the oral polio vaccine, causes of “resistance” to vaccination, confidence in the program’s ability to succeed, and the provision of health services. Two translators who were familiar with the local environment, fluent in local dialects of Hindustani (Hindi, Urdu, Bhojpuri, and colloquialisms from Eastern Uttar Pradesh where some migrants had arrived from), and trained to conduct health promotion field activities joined the researcher in conducting interviews. All interviews were conducted in either Hindustani or English by the researcher with the assistance of the translators.

In total, 107 structured or semi-structured interviews were conducted. Participants included 80 families with children in the aforementioned parts of Aligarh, and 27 stakeholders in the eradication program. Each structured interview took 30–40 minutes, and was conducted at a site of the interviewee’s choosing. Transcripts were either recorded by hand or with an electronic recorder with the permission of the interviewee. All interviews were made confidential unless the right was specifically waived. Interviews were conducted to the point of saturation, as data repetition occurred at all levels, indicating the views found reflected that of a substantial portion of the studied respondents [35].

### Data Analysis

Analysis of the data was conducted by the researcher independently. All transcription and translation was done by the researcher who is fluent in the Hindustani dialects of Hindi and Urdu. Due to difficulties with sound quality and local dialects such as Bhojpuri, full transcription was conducted for 20 of the recorded interviews. Partial transcription was done for the remaining 82 recordings in addition to the interview notes taken during all structured and semi-structured interviews which aided in deciphering with the assistance of translators during that portion of the study. Of the structured and semi-structured interviews, 5 had written interview notes exclusively. All informal interviews were written alongside field notes. No software was used in the analysis of the data, which was manually coded for causes for “resistance” in the Muslim community, opinions about the causes of segregation and differential development in Hindu-majority versus Muslim-majority areas, behavior of the polio vaccination teams, and trust of the medical establishment and government. This coding scheme was developed based on previous literature about causes of “resistance” and inductions from the participant observation. The informal interviews from the field notes and transcripts from the interviews were coded and grouped by the described major themes to give a better understanding of “resistance.” Data from participant observation, semi-structured interviews, and formal interviews based on active recruitment were given equal weight and not differentiated during analysis.

## Results

### Riots and Segregation

**Fig 1:**
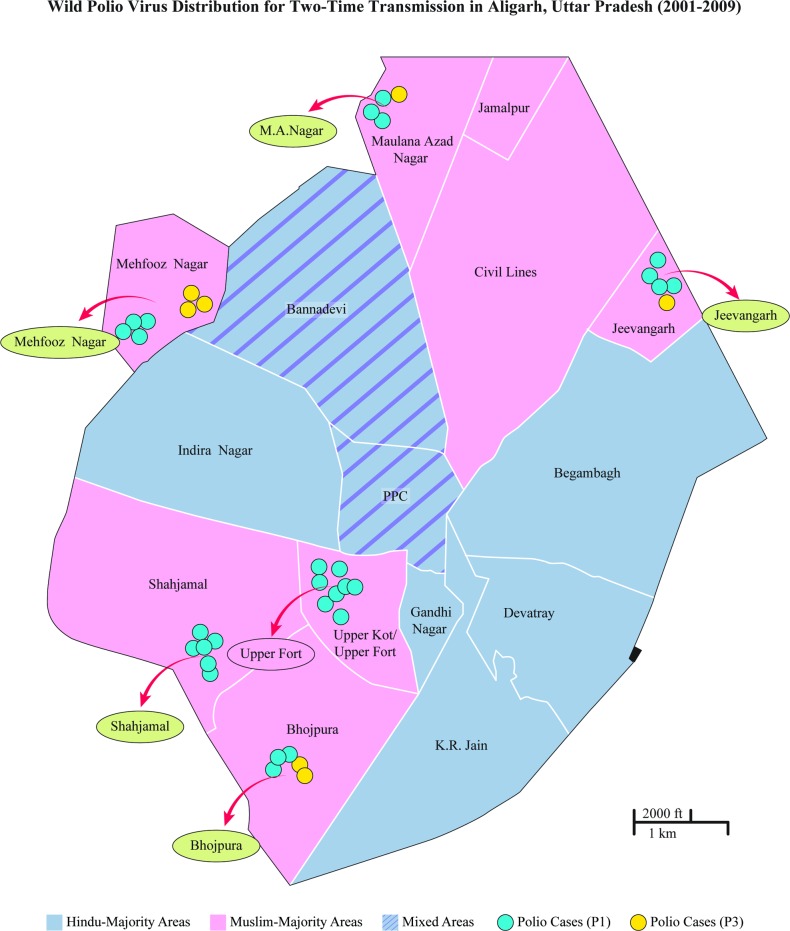
Qualitative mapping of Aligarh’s segregated neighborhoods with National Polio Surveillance Project (NPSP) “two-time transmission” cases (2001–2009). Ward distinctions based on maps used by NPSP [2].

**Fig 2:**
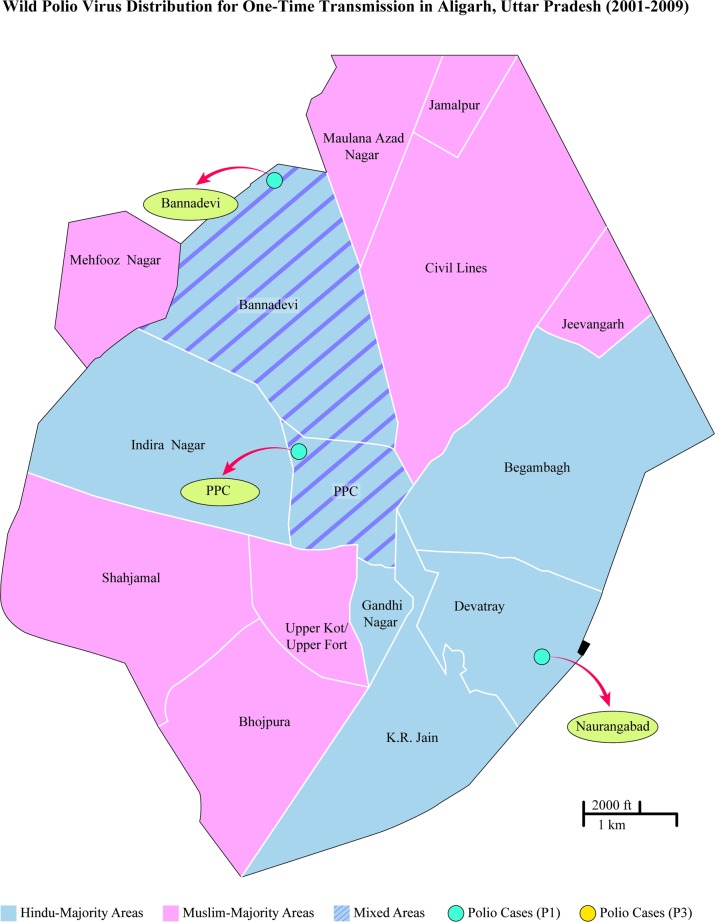
Qualitative mapping of Aligarh’s segregated neighborhoods with National Polio Surveillance Program (NPSP) “one-time transmission” cases (2001–2009). Ward distinctions based on maps used by NPSP [2].

Almost all respondents who participated agreed that Aligarh was a highly segregated city along religious lines, and that the segregation occurred because of riots after Partition and during the 1990s. Most of Aligarh was classified by locals into “Muslim areas,” “Hindu areas,” and “Mixed areas” (see coloration, Fig. 1 and Fig. 2). A resident of Shahjamal, one of the Muslim-majority slums, said that his neighborhood used to be mixed but after the destruction of the Babri Mosque, everyone separated. He explained that the land from the “Dargah” to Khair Road once had a significant Hindu population, but was now predominantly Muslim. The opposite side of Khair Road, had large pockets of Muslims such as in “Basani Ka Nagla,” but after the “martyrdom of Babri Mosque,” Muslims and Hindus fled to areas where they formed a religious majority. Driving down Khair Road, the boundary between Shahjamal and Indira Nagar, this segregation is apparent as one side of the road has several temples and a very large Hindu population whilst the other side is dominated by Muslims.

Respondents in the wealthier Civil Lines, usually spared from riot violence, explained that faculty at the AMU began distrusting one another and ceased to live together around the same time. One older gentleman who lived near campus explained:

...No it has never been like this...Indian history will be written....post-Independence Indian history...will be written....post-Babri Masjid and pre-Babri Masjid demolition. Things were very nice till December 1992 when Babri Masjid was demolished...by some fanatics...but that led to riots all over India...and people...Aligarh...suffered many riots following that till 1994...people started feeling unsafe...and this migration of the population has occurred...post 92

I will give example [sic] of our medical colony. All non-Muslim and Muslim teachers were staying together in Medical Colony. Post-92' those who are our friends, we have always been with them, they even migrated there in spite of the fact we gave them full protection. The vice-versa was also true, the Hindu population also gave protection but the people were not going to believe them because they were afraid of those goondas [criminals] or that element which is there which can cause harm to them. That is why this segregation was initially rapid and then gradually everyone sold their property there and came to Jamalpur and Jeevangarh... (newer Muslim-majority developments near the university)

Though some people felt that Hindus and Muslims had lived in a separated manner for generations, the vast majority of respondents indicated it was a recent phenomenon, pointing to riots as the principal cause. Many respondents were displeased that everyone lived separately, and made a point to say that communal relations were much better today than they had been in the recent past. Nonetheless, almost all respondents seemed content with religious isolation due to fears of the previous riots and commonalities in ritual and practice.

### Differential Development of Segregated Slums

Since the formation of segregated neighborhoods, almost all respondents described that “Muslim areas” had been underdeveloped compared to “Hindu areas.” There were two dominant theories regarding this development: that the government provided services for Hindus but not Muslims, or that Muslims had poor leaders and did (or could) not take advantage of the political system.

Most interviewed Muslim slum dwellers believed that their areas were ignored by the government because they were, in fact, Muslim. In an earlier part of the project when the question of differential development in Muslim areas had not formed, one male Muslim respondent in Jeevangarh responded to a generic question about whether he felt the government was performing its duties, “What's the government doing? It’s not doing anything for us. There are no services here. Just for the educated and those who are connected to them. There are no services here. There are no services in any of the Mohamadan (Muslim) neighborhoods.” Highlighting their neglect compared to other areas, a family residing in Shahjamal explained that they thought the “Hindu government” took care of Hindus, whereas the Muslim politicians did nothing for them. As the mother in the family explained:

Some places are such that, like look at the place in front of you...is completely flooded...and it hasn't even rained...and when it rains...the water will be up till here [points to top of stoop]. This is a neglected area. In some good neighborhoods, treatment is done and the government works on things immediately...If it is a Hindu government it will look after Hindu homes. Even when we call a politician to come and look after something, (he will do so) only after a month, two months, or a year. Then he'll think about it. Then if the budget is available he might do something. Otherwise he won't do anything. The politician may be a Muslim but even then he won't do anything.

Most poor slum-dwelling Muslims harbored this negative view of the politicians. Some low-caste Hindus shared it as well. For example when a Scheduled-Caste member in the slum-area of Kishanpur was asked about differential development in Hindu versus Muslim areas, he took the team into the street and pointed out how all the roads in his neighborhood had recently been paved with cement, save the one Muslim-majority street. The narrow Muslim gulley had a dirt road and an open sewer that had already started to flood. Both poor Muslims and Scheduled Caste members often responded to questions about differential development by telling the team to “just go take a look yourself and tell me if the government is biased.”

Wealthier Hindus and Muslims usually agreed that Muslim areas were neglected by the government. However, they felt Muslim areas were neglected not because of outright discrimination, but because Muslims had few political representatives. One Muslim respondent from Sir Syed Nagar explained that Muslim areas were “definitely” less developed compared to non-Muslim areas because Hindu politicians often knew they would get little political support from the Muslims. He explained:

The government's apathy is there. The government generally doesn't want to develop these (Muslim) areas. They know they don't get the votes from these areas so they will only develop those areas from which they get the votes. That is the assumption they have, the local leaders. They don't think they are leading the whole community, they think they lead only those areas from which they are getting the vote, so that leads to discrimination.

One female respondent who worked as a seamstress in the Civil Lines neighborhood of Dhodpur agreed, explaining that Muslim areas were less developed than non-Muslim ones because they had no strong leaders, as most remained impoverished, limiting their power:

Why like this (underdevelopment in Muslim areas)? Because amongst [sic] Muslims there are no good leaders or representatives. They don't have enough money to pursue leadership and get stuck just trying to feed their families. Hindu families are established, they have leaders.... (undecipherable)...It is like this, upon nominating their representatives, they get development work done personally. Muslims are less educated so they are not able to get their work done personally.

Comparatively, non-Muslims were perceived to not only be better off, but much more politically connected, allowing them to develop their areas. The same individuals interviewed above explained that Hindus were wealthier, had successful businesses, and had their needs met by the politicians. She explained, “It (the condition of Hindus) is better than ours. Good... They have business and are resting in peace. Nobody bothers them, any leader etc. For Muslims they come and disturb and tell us to do this work or that work help those people.” Though the condition of poorer Hindus and Dalits was also abysmal, people believed they had stronger governmental representation and better infrastructure in their neighborhoods. Both poorer and wealthier Muslims shared the impression that even poorer Hindu areas were “better off” than economically comparable Muslim areas. A middle-class professional man from the Civil Lines (who, it should be noted, did seem to exhibit some biased opinions regarding communal affairs) explained, “With the Hindu community, even if they are impoverished...generally their areas are cleaner. Muslims are living in areas which can hardly be considered clean...they are living in holes which are filled with water...there is filth everywhere.”

Because of this slightly improved development, one gentleman in Sir Syed Nagar said that compared to Muslims, the poorer Hindus were more likely to accept the polio vaccine than Muslims who “resisted” due to the lack of development:

The different neighborhoods are divided largely into segments of different communities. Dalits reside in Jamalpur (near campus), and in the outskirts of Jeevangarh and Mehfooznagarh. The conditions of these communities have improved generally because of government emphasis on their community in recent years because they were historically marginalized. They accept the vaccines now, are less resistant, though the case is not so in these communities (the Muslim ones).

### Resistance and Facilitated Spread of Polio

Respondents felt that the neglect of Muslim slums facilitated further distrust of the government and corresponding resistance to vaccination. One male clinician in Jeevangarh explained: “If the government had not done any ‘good’ for them before this point, then how could the people be expected to trust the government now?” One of their colleagues agreed, when asked:

Researcher: Some people say that a lot of people are XR (resistant to vaccination) because work...development...does not happen in their areas. They say the government doesn't do any work there.

Respondent: Yes! This is also true. First of all they don't have jobs which is their biggest concern. If they don't have jobs they don't have food to eat. And they aren't getting services...the gullies ahead of them are bad, the sewers are bad, they don't have light (electricity), or even water. They are worried in every sense of the word, and on top of that when the government comes and tells them they are doing this for their benefit, that it is doing good work to protect them, then from within, their frustration comes out that if they are doing it for their benefit, why aren't they doing anything else...why just polio...their children are starving to death and they don't have money for medical treatment. Why aren't they getting the medicine that they need? So this is true...because they don't get any services, they become XR.

In fact, there was generally a prevalent view that families, particularly Muslim ones, were taking the polio program and turning it into a “weapon” to bargain and get development done in their areas. As one male stakeholder explained:

And another thing, because there has been no development in those areas where Muslims are living, they have made this program a bargaining point that if you want to give polio vaccine to our child, then you will have to provide roads, sewage facility, drinking water etc. to our areas. So it’s somewhat of a bargain going on between the community and the government.

Additional resistance stemmed from the fact that families felt the lack of infrastructure allowed the spread of disease, including polio. Though the majority of participants did not know how to explain disease etiologies, many were sure that gandhagii (filth) was a cause of illness. An uneducated mazdoor (laborer) from Shahjamal, answered a question about etiology:

Researcher: But first a more general question, this polio disease, in your view, how does it spread? Do you know? You can say what you feel. There is no correct or incorrect answer.

Respondent: The sickness here. The filth...the government does not stop it. If only the filth could be finished off.

A female polio vaccinator who worked in the largely Muslim “high risk” areas further explained:

Polio Vaccinator: There is so much filth that all the sewers are open. And all the small sewers go towards the larger one. When it rains, the larger sewer boils up and floods onto the streets where the children are wandering. Of course, disease is going to spread.

Researcher: Not just polio but other...Polio Vaccinator: Not just polio, other diseases also, but polio too. First you have to improve the infrastructure, and only then will the disease load be reduced.

Many of the children visited by the teams on the polio rounds were found to be constantly sick, suffering from respiratory infections, diarrhea, parasites, worms, fevers, or other ailments. Consequently, many of the families were resistant because they were afraid to vaccinate their already weakened children. Such families were labeled “XS,” that is, resistant due to sickness. Expressing her frustration, one mother in Jeevangarh screamed:

I think the polio medicine is useless...yeah. When my girl is sick they force us to drink the medicine. They call the doctors and the police on us and forced us to vaccinate. We have malaria here...I tell them not to come to my door...we are in so much misery...the children have diarrhea and you are forcing them to take this other medicine...They are taking sick children and forcing them to drink the polio vaccine...If the child is sick make the child feel better first!

An anonymous interview with one GPEI official also emphasized resistance in the Muslim community. “The data doesn’t lie,” he said, pointing to a graph indicating that in 2008, 53% of P1 cases were in the Muslim community, disproportionate to their population of 18% in UP. Since the eradication strategy had used the monovalent mOPV1 on polio rounds since 2007, targeting only the P1 strain of the poliovirus, this disparity indicated that the Muslim population is resisting vaccination, he said.

The data also indicated that Muslims were disproportionately affected with P3 relative to their population, though it was spreading “naturally.” When asked why 16% of the population comprised 34% of P3 cases if P3 was spreading “naturally,” he responded that “other determinants” were the cause. Pointing to cases in Bhojpura and Mehfooz Nagar on a map of Aligarh, he explained, “Cases largely occur in slums on the outskirts of the city. In intermittent years they might travel through Hindu neighborhoods and we might get a few cases among the Scheduled Castes, but these areas are surrounded by wealthier Hindu areas with better infrastructure, which protect them.”

This data is seen more explicitly with qualitative mapping of “Hindu majority,” “Muslim majority,” and “Mixed” areas from interviews, observations, and rapid surveys. The demographic breakdown of these wards by religious community has been substantiated by other researchers as well who highlight the sharply demarcated segregation of these neighborhoods. The polio-case data by ward was provided by GPEI partner organizations. These figures reveal that two-time transmission polio cases of both types occurred disproportionately in “Muslim majority” areas (see Fig. 1). Conversely, one-time transmission occurred in what were mapped to be “Hindu-majority” or “Mixed” areas (see Fig. 2).

### A Rumor Contextualized

Though the primary causes for social “resistance” to vaccination were regarding development and illness, some continued to be based on distrust of the local government. Though much of the media attention on the polio outbreak focused on a conspiracy theory that Western governments were sterilizing Muslims, from all 107 interviews conducted and polio rounds observed, only three respondents identified or even mentioned a Western plot. Only one of these believed in the plot. At the time of this study, people who feared the vaccine might sterilize their children in Aligarh were more likely to blame the government, polio workers, or flaws with the vaccine rather than an American or Western plot against Muslims. When speaking with reference to causes of the polio disparity, a prominent physician in Aligarh who had been working with the eradication program said on condition of anonymity:

... (a) point which comes to my mind is that when the program was started, it was started by a central government which was...in inverted commas 'not friendly’ to the...to the community. And this issue was politicized by other parties, who were against the ruling party at that time...and they said they are trying to give this medicine, this immunization again and again so that the fertility of the Muslims, combination is...will become less and they are not able to produce children in the future...

Thus, though he did not acknowledge or believe that the central government at the time did anything hostile to the Muslim population, rather blaming opponents of the Hindu-right for exploiting the situation, he acknowledged that the Muslim population feared the polio program because it was started under a government ‘not friendly’ to the community.

Since the GPEI cooperated with local governments, during the course of the study, this link caused immediate hesitation amongst some individuals. Study participants generally labeled both GPEI agencies and the Indian government as the sarkar (government), and the polio program a “sarkari program.” Other than in educated circles where there was widespread knowledge about different international agencies, the GPEI and government were seen as one and the same.

This mistrust was also seen with the politics of fertility. For example some Muslims in Aligarh believed that they were specifically targeted for sterilization during the ‘Family Planning Program.’ A community physician who worked in Aligarh’s Muslim-migrant slums explained in a conversation:

Respondent: See, because there is a program of forced sterilization, 1977, that led to topple of Indira Gandhi government...and Janata party came into power...and at that time there was a word called ‘Family Planning Program’ and that was due to this factor that this (the name) was changed because family planning created a resistance, especially amongst the Muslim community. Researcher: Only in the Muslims?Respondent: It was there in everyone, but largely among the Muslim sector there was forced sterilization. People were put in jail. So that program was changed to family welfare program. Planning was...as soon as somebody spoke out ‘Family Planning’ the bells that rang into the mind was of sterilization. So that might be the case, people must have thought actually they were trying that way, and that way didn’t succeed so this one might be the case.

However, most interviewees initially responded with either hesitation or confusion when asked about the ‘Family Planning Program.’ Most physicians, clinic workers, and health professionals believed the history had a significant impact on the spread of the sterilization rumors. One Muslim respondent and his wife in the urban slum of Shahjamal indicated that the history of Sanjay Gandhi’s ‘Family Planning Program’ showed them that there was a continuous initiative on the part of the government to sterilize Muslims. They believed the polio program was simply a “reincarnation,” or a new manifestation, of the ‘Family Planning Program.’ When asked if they thought the sterilization rumors associated with the polio program were linked to this history, they responded:

Respondent: Yes, from that time which is why we are being attacked by polio (program) [sic]. Translator: He means that before they had done forced sterilization, now the government is not doing this, but doing this through the polio programRespondent’s Wife: So that there can be no future generations. Researcher: So if the government did it before, you feel it will do it again? Respondent: Yes yes. Respondent’s Wife: That's exactly how we feel. That same program is repeating. It is happening in another guise. In another life. The program will change its nature. It will be reincarnated in another form.

## Discussion

The results of this rapid ethnographic research reveal insights into how the status of Indian Muslims as a sociological minority contributed to a significant polio disparity since at least 2002 [2, 36]. Heightened awareness and sensitivities regarding communal identity in post-Partition India facilitated disharmony, conflict, and promoted the formation of segregated slums. The polio disparity is best understood in this historical context as it led to the development of structural violence. The manifestations of structural violence elucidated from this study include fears based on historical realities, distrust of government and GPEI workers, and perceived discrimination and deprivation in Muslim slums. Respondents explained their concerns about the slums as disease-spreading environments, which would be accurate due to the oral-fecal transmission of the virus and documented higher attack rates in slums, including particularly North Indian Muslim slums [37, 38, 39]. Many demonstrated resistance to vaccination on grounds varying from a sense of misalignment of priorities with a vaccine ostensibly offered for their benefit when other services were notably neglected, to also fatigue from the repeat doses of vaccination described previously [40]. These experiences fueled social resistance to the polio program with heightened risk perception (e.g. beliefs that the vaccine was tainted or that children were already too sick) and lack of trust regarding power relations with the state (e.g. skepticism related to Pulse Polio Immunization (PPI) as a government program and concerns about state population control).

Previous studies support our findings with regards to specific factors contributing to the polio disparity. The already manifest disparity gained media attention in 2006 after the spread of the “sterilization rumor,” exacerbated the situation, with an almost exclusive focus on the rumor as the cause of the disparity [41]. Since that time, a more nuanced understanding has been deciphered and factors including poor sanitary conditions [42], suboptimal OPV effectiveness, particularly in underdeveloped slums [43], lack of public awareness [44], and non-inclusive health systems [45], have been found to contribute to the disparity. Though the nuances of the surge have been deciphered in both the Indian and most prominently the Nigerian cases (where the rumor originated) [46], few studies have explicitly focused on how these factors and the status of Muslims as a sociological minority together contributed to the disparity [47, 48]. The results of our study place each of these findings within their historical context and synthesize the multiple etiologies of the disease disparity under an overarching theory.

One of the primary contributors to the disparity was the development of ethnic slums. As seen with mapping the National Polio Surveillance Project (NPSP) polio data with our qualitative assessment of the neighborhoods (which has since been corroborated quantitatively regarding the demographic breakdown of each ward [10]) polio cases were concentrated with higher transmission rates in Muslim enclaves, specifically Muslim slums. Almost all respondents in the study agreed that riots after Partition and during late 1980s and early 1990s with the rise of Hindu nationalism were the principal causes of segregation. They also agreed that segregated Muslim-majority areas generally had poorer infrastructure than Hindu-majority areas. This was also a general conclusion found in the Sachar Committee Report.

“... Compared to the Muslim majority areas, the areas where fewer Muslims inhabited had better roads, sewage and drainage, and water supply. Often there was a school and a health centre which were absent in areas where Muslims of similar economic background had a large share in population. For instance, a Hindu-dominated urban slum in Lucknow had better quality roads, drainage system, sanitation, water supply and sewage disposal compared to another slum populated largely by Muslims” [13].

Though the cause of underdevelopment is not confirmed, Muslim disenfranchisement in education, politics, employment, and economy, are like contributors. This is highlighted by respondents’ views on the lack of Muslim political power as described above. Muslim political weakness was widely seen as a consequence of post-Partition withdrawal of Muslims from politics, especially as the educated middle class had left for Pakistan. This was especially true in Northern States such as Rajasthan, Uttar Pradesh, and Bihar due to their proximity and the Muslim League’s success in these regions amongst the Muslims. Recovery has been stifled in this region since that tumultuous era [13, 18]. Some respondents believed that modern Muslim leaders were themselves poorly connected and unable or unwilling to participate in the political system, depriving their communities of a way forward.

Notably, class differences amongst the Muslims in Aligarh were especially sharp, and the political empowerment of individuals connected to the Aligarh Muslim University did not extend to the Muslims who lived in the slums. As mentioned, many Muslims who lived on the “Civil Lines” side of the city where the university was, rarely if ever went to the old city, let alone one of the Muslim slums. Rather, the presence of the University and its historical connection to the Pakistan movement has been used by communal forces to instigate riots against poorer Muslims in the past [11, 16].

The underdevelopment of Muslim areas appeared to contribute to social resistance to vaccination. Lack of development has been known as a general cause of resistance as people refused vaccination due to perceived misalignment of government priorities: failure to provide basic infrastructure while compelling individuals to vaccinate [47, 49]. Vaccination campaigns like the polio program are particularly vulnerable to the proliferation of rumors simply because they can be both intensive and disruptive, causing people to question the benefits of programs [50]. A detailed explanation of the social causes of resistance to vaccination in the Indian context and vaccine acceptance behavior by involving the affected communities is outlined in a previous article published from this study [40]. These included fatigue from repeat dosages of the medication, frustration from the lack of basic health services, distrust of the efficacy and safety of the vaccine (including the possibility of paralytic disease from VAPP or Vaccine-Associated Paralytic Poliomyelitis and VDPV or Vaccine-Derived Polioviruses), and the lack of true informed consent with obtaining the vaccination due to the incomplete training of some of the gate-keepers who delivered the vaccine. Similar distrust and resistance has occurred in Nigeria’s neglected Northern areas due a sense of mistrust of the mostly Southern-based government and distrust of Western medicine in light of the failure of an antibiotic for meningitis [39, 51, 52]. This was in addition to fear of the vaccine’s safety and efficacy and the spread of the sterilization rumor itself [51, 52]. Muslims’ perception of an exertion of state power, combined with skepticism understood in the context of historically-rooted deprivation and socio-political exclusion, appears to exacerbate resistance from these more general causes leading them to believe the vaccines were ostensibly offered for their benefit. Poor infrastructure in the slums also led to the aforementioned higher physical attack rates with the poliovirus spreading via the oral-fecal route, and has caused lower vaccine efficacy rates from both competing enteroviruses and diarrheal diseases which flushed the vaccine from the body [37, 38, 39, 43]. Low vaccine efficacy necessitated a higher intensity dosage regimen in Muslim areas compared to non-Muslim areas which further fueled social resistance [40, 44].

As described by a polio program stakeholder, social resistance to vaccination could also be used as a “bargaining point” for obtaining better services. This finding is supported by previous ethnographic studies of social resistance to polio vaccine in Uttar Pradesh [47, 49]. The notion of resistance as a negotiating tool was also seen among Muslim groups in this area during the smallpox eradication campaigns, demonstrating a continued problem which would need to be addressed for future eradication campaigns [53]. Polio vaccine boycotts have also been seen in Nigeria to demonstrate how participation became a leverage point for negotiating better access to basic services [54].

Thus it would appear that physical violence from both the Partition era and from the rise of Hindu Nationalism in the 1990s led to the development of segregated slums. In light of poor leadership or other causes, these slums became underdeveloped compared to economically equivalent Hindu slums. This lack of development and poor infrastructure led to the physical spread of the virus in these communities, with higher polio attack rates in addition to other illnesses. Some of these illnesses flushed the vaccine and reduced the efficacy of the vaccine. The spread of other illnesses would mean that the Muslims had sick children more often and would also be likely to refuse vaccination on these grounds. In the setting of lower vaccine efficacy, additional doses were needed which made the Muslim population more likely to resist the vaccine due to fatigue and a sense that it was emphasized in manner more directed at their community. Since Muslim areas were perceived to be neglected and have poor health services (and amenities in general), there would be more skepticism about services which seemed especially targeted towards them, enabling “bargaining” behavior for the vaccines in addition to allowing an easy proliferation of rumors in light of previous distrust due to historical realities.

Historical fears related to the “Family Planning Program” and the anti-Muslim sentiments voiced by Hindutva politicians appeared to reinforce Muslim distrust of the polio program, fueling social resistance to vaccination campaigns. Unfortunately, as recently as March of 2009, Varun Gandhi, Sanjay Gandhi’s son and a member of the Hindu nationalist Bharatiya Janata Party (BJP), proclaimed at his rallies that the government ought to “pick them (Muslims) up, one by one, and sterilize them” [55]. Individuals in Aligarh saw the polio program as a ‘sarkari’ or government program, and felt the government was capable of forcibly sterilizing its own people. Though the sterilization rumor originated in Nigeria and initially pertained to a Western plot, because of this historic tension, when the misinformation arrived in India, it was quick to spread. Most health professionals in our study believed this history had a significant impact on the perpetuation of sterility-related rumors about polio vaccine. This historic distrust can be likened to the US government’s “Tuskegee Study of Untreated Syphilis in the Negro Male.” In the study, poor black men with syphilis were recruited with the promise of care, only to be left untreated until autopsy. The deceptive recruitment and withholding of care fractured African-Americans’ faith in the medical system in a way that is yet to recover [56]. From this study, it seems that India’s ‘Family Planning Program’ may play a similar role for India’s marginalized communities.

These results are supported by previous findings linking Muslim resistance to vaccination with fears related to India’s historical Family Planning Program [57]. It should be noted that the Family Planning Program was conducted under the auspices of a secular political party nationally, and that Muslims remained skeptical of both secular and Hindu nationalist political parties. Such continued skepticism will be a barrier which needs to be addressed for public trust in health promotion campaigns in the future [58, 59]. To do this, these fears and rumors must be discussed and addressed publicly, with an effort to understand and address them. According to Leach and Fairhead, such rumors could make sense from standpoints “which link individual weakening with the weakening of the body politic, as the population or area is sapped of fertility and strength” [60], highlighting the need for them to be understood and addressed.

The reasons for polio vaccine resistance among Muslims in India are best understood by taking into account the historical and social context in which the program was implemented and using a multi-pronged explanatory perspective [28]. Muslims’ minority status and the physical violence inflicted against them after Partition caused the formation of segregated Muslim slums and made Muslims victims of structural violence. These experiences impacted vaccine acceptance through influencing trust and perceptions of risk, and impacted physical risks with facilitating the spread of the poliovirus and decreasing vaccine efficacy. Thus, explanations for suboptimal vaccine acceptance among Muslims blamed on “stubbornness” or “radicalism” obscures the reality that vaccination campaigns are sociopolitical phenomena that incorporate both historic and ongoing conflicts [28]. The bodies of Indian Muslims in Uttar Pradesh and regions where the polio disparity was present can be seen as embodiments of their social marginalization where they have been victims of physical and structural violence, manifesting in the polio disparity.

### Looking Forward

In order to solve the problem of polio in India, UNICEF reached out to neglected communities—including Muslims—by gaining the support of local leaders and providing basic services through the “Underserved Strategy” [37] [61]. The information, education, and communication activities undertaken by the Social Mobilization Network (SMNet) allowed Muslims and other disadvantaged communities to have a stake in the eradication program, build bridges, and provide other medical supplies to show the community that their overall health needs were considered [61–63]. These intensive efforts have resulted in a remarkable drop in the disparity with Muslims bearing over 50% of cases from 2002–2007 to 38% in 2008, and a continued trajectory of declining polio cases in India [64], culminating with the declaration of India being polio-free in March of 2014[65]. Nevertheless, the lessons of this study are relevant for future efforts related to keeping India polio-free, and translating these lessons to both other health problems in India and comparable situations in the remaining polio-endemic nations [66].

The spread of “social resistance” in each of these nations has its own local historical context, nature of spread and should be addressed as such. In Afghanistan and Pakistan, negative sentiment toward the local government, international agencies, and the continued insurgency and war constitute different challenges for eliminating social resistance and for protecting polio workers than in India. Nevertheless, the successful efforts to combat polio amongst disenfranchised Muslims in India ultimately suggest that this is possible in other nations and can be translated to address social resistance there. In Pakistan there has been distrust of the vaccination program due to the CIA’s use of a Hepatitis B vaccine in order to procure DNA to find bin Laden [67, 68, 69]. This has often proliferated as a rumor that the CIA had used a polio vaccinator to find him, and has even led to the tragic death of some polio-workers who were attacked and even killed by resistant families [70, 71]. Much like the disenfranchised Muslims requested services to “bargain” for polio, the Taliban has attempted to “bargain” to allow polio workers vaccinate if drone strikes are stopped [72]. This is a dangerous development and will need to be addressed in a way uniquely appropriate to the situation. Nonetheless, understanding the historical context, the discussed factors of structural violence, and the way in which they interacted and contributed to disproportionate spread can inform future strategies for India as it transitions toward creating new policies to prevent re-introduction of the virus, as well as to target the virus for eradication in the remaining three nations [73].

Our analysis of the polio disparity in India also adds to the small, yet growing body of literature on Muslim health inequalities in India. The Sachar Committee Report was the first of its kind to analyze the systematic deprivation of Muslims across multiple social indicators, and few studies have examined health disparities among these groups. It has been documented that Muslims experience lower rates of child immunization [74] and higher scores of child health inequality measures related to immunization coverage, stunted growth, and being underweight [13, 75]. Lower utilization of safe maternity care services have also been documented among Muslims in India [76], and long term mistrust of government has been cited as a factor in understanding this resistance to new policy initiatives. The authors stressed the importance of improving broader level institutional services in order to increase acceptance to these more targeted health promotion efforts [77]. Furthermore, a study of pandemic preparedness in India concluded that Muslims are at risk for being disproportionately burdened by an influenza pandemic and concluded that more robust primary healthcare services are essential in averting such a disparity [78].

The findings from our research point to the urgent need for solutions that take into consideration how structural violence contributes to health disparities among Muslims in India, particularly in the North of the country. They suggest that future health promotion efforts among this group need to address and rectify broader social and economic inequalities among Muslims. More research needs to be done in this area in order to ensure that particular attention is paid to the most vulnerable, both in future global efforts to eradicate polio as well as efforts to improve health and reduce disparities in India.

## Limitations

Due to the manual coding of the transcribed data and field notes, this study may be subject to researcher's bias. Selection bias may have occurred due to a high frequency in declination to interview among unspecified potential participants in addition to the smaller sample size generally. Additionally, as not all interviews were completely transcribed, the full scope of views shared by participants may not have been acknowledged. Difficulties translating local dialects and poor sound quality may also hinder analysis of the data. Nevertheless, the study provides insight into views and attitudes toward vaccination which were prevalent at the time of the study.
